# Improving the Assessment and Management of Cardiovascular Risk in Adult Psychiatric Inpatients Using the QRISK3 Score – A Combined Quality Improvement and Pilot Study

**DOI:** 10.1192/bjo.2025.10332

**Published:** 2025-06-20

**Authors:** Ahmed Al-Shihabi, Alex Berry

**Affiliations:** North London NHS Foundation Trust, London, United Kingdom

## Abstract

**Aims:** Individuals with severe mental illness have been recorded to have a life expectancy 10–20 years shorter than the general population, with part of this discrepancy being attributable to an increased risk of cardiovascular disease. The QRISK3 score is a validated tool for assessing an individual’s 10-year risk of a myocardial infarction or stroke.

Our aim was to assess the practicality and impact of making calculation of the QRISK3 score routine practice for new admissions onto our general adult acute male inpatient ward, in order to improve detection of increased cardiovascular risk and offer atorvastatin as primary prevention if indicated.

**Methods:** Over the course of six months (August 2024–February 2025), we calculated the QRISK3 score for 50 inpatients on a general adult male acute ward. Patients who had a score of 10% or more were counselled on their increased risk of stroke or myocardial infarction, and were offered atorvastatin as primary prevention.

**Results:** At the start of data collection, only one of the 17 patients on the ward was on a statin and none of the patients had a documented QRISK3 score.

Of the 50 patients included, 10 of them had a QRISK3 score of 10% or more. Of those 10, two were already on a statin. Of the remaining eight, four agreed to start atorvastatin whilst the remaining four declined.

QRISK3 scores were included on the discharge summaries of all patients who they had been calculated for, with a request to the patient’s GP to revisit the topic of primary prevention in the future for those patients who had declined a statin.

The average time to acquire the information required to calculate the score for a patient was 6 minutes and 24 seconds.

**Conclusion:** Calculating the QRISK3 score for psychiatric inpatients is a quick process that can feasibly be a part of a checklist for new psychiatric admissions and may increase the proportion of patients on appropriate treatment with a statin.

In the future, use of a semi-structured interview that includes both statin counselling and lifestyle advice can be implemented, and we will trial this for the second cycle to see if it has an impact on uptake of a statin. Future research could involve longitudinal follow-up of cardiovascular outcomes to assess the impact of primary prevention in this patient population.

